# Evaluación de la prueba fecal Liaison^®^ Calprotectin de DiaSorin adaptada al derrame pleural

**DOI:** 10.1515/almed-2023-0148

**Published:** 2023-12-04

**Authors:** Cristina de Paz Poves, Clara Barneo-Caragol, Ana Isabel Cillero Sánchez, Lucía Jiménez Mendiguchia, Covadonga Quirós Caso, María Moreno Rodríguez, Francisco J. López González, Mª Belén Prieto García

**Affiliations:** Laboratorio of Medicina, Departamento de Bioquímica Clinica, Hospital Universitario Central de Asturias, Oviedo, Asturias, España; Departamento de Bioquímica Clinica, Hospital Álvarez Buylla, Mieres, Asturias, España; Departamento de Bioquímica Clinica, Hospital Universitario San Agustín, Avilés, Asturias, España; Departamento de Neumología, Hospital Universitario Central de Asturias, Oviedo, Asturias, España

**Keywords:** automatización, calprotectina, quimioluminiscencia, líquido pleural, validación

## Abstract

**Objetivos:**

La calprotectina (CP) es una proteína de unión a calcio y zinc que se suele determinar en muestras fecales, aunque su cuantificación en otros fluidos biológicos podría ser de interés. El objetivo del presente estudio es validar la determinación de CP en líquido pleural mediante quimioluminiscencia.

**Métodos:**

Para la cuantificación de CP en líquido pleural, se utilizó LIAISON^®^XL, un autoanalizador de quimioluminiscencia. Se diseñó un protocolo de validación empleando tanto materiales de control de calidad suministrados por el fabricante, como soluciones de muestras de líquido pleural. Se evaluaron la estabilidad, imprecisión, sesgo, linealidad, capacidad de detección y efecto de arrastre.

**Resultados:**

La CP permaneció estable en líquido pleural refrigerado durante al menos una semana, y durante cuatro semanas a −80 °C. La imprecisión intradía e interdía observada fue del 2,2 % y del 6,49 %, respectivamente, con un sesgo negativo del 5,51 %. La linealidad del método se verificó hasta los 2000 ng/mL. El límite de cuantificación (LoQ) de la prueba fue de 48,52 ng/mL. Se observó un efecto de arrastre estadísticamente significativo tras medir concentraciones de CP superiores al límite máximo de linealidad. Sin embargo, dada la magnitud observada, no se debe esperar un impacto clínicamente relevante.

**Conclusiones:**

La prueba Liaison^®^ Calprotectin de DiaSorin es fiable para la determinación de CP en líquido pleural.

## Introducción

La calprotectina (CP) (S100A8/S100A9) es una proteína de unión a calcio y zinc de 36 KDa, que pertenece a la familia de las proteínas S100 [1]. Esta proteína, principalmente producida por los neutrófilos, se encuentra elevada en procesos inflamatorios, siendo un indicador de quimiotaxis de los neutrófilos [2].

En la práctica clínica, la determinación de CP fecal [3] se suele utilizar en el diagnóstico y seguimiento de pacientes con desórdenes gastrointestinales inflamatorios crónicos [4], aunque también se puede determinar en otros fluidos biológicos [5], como el derrame pleural. Estudios recientes han señalado su utilidad clínica a la hora de predecir malignidad con precisión, aunque en todos se empleó la técnica ELISA [6], [7], [8] que, entre otras limitaciones, no es adecuada para pruebas que requieren tiempos cortos de respuesta.

Sin embargo, actualmente disponemos de métodos totalmente automatizados, rápidos y fiables para la determinación de CP. El objetivo del presente estudio era validar la determinación de CP en líquido pleural mediante quimioluminiscencia, bajo la hipótesis de que este método mostraría mejor rendimiento clínico que ELISA.

## Materiales y métodos

### Principios de la prueba e instrumento

Se utilizó LIAISON^®^XL (DiaSorin, Italia), un autoanalizador de quimioluminiscencia, para la cuantificación de CP en líquido pleural. Este analizador fue originariamente diseñado para la cuantificación de calprotectina fecal, por lo que presenta los resultados en µg/g. Basándonos en un estudio previo [9], utilizamos un factor de conversión de 2,5 para transformar dichas unidades a ng/ml, para las muestras de líquido pleural. También se transformó la curva de calibración, siendo el rango de medida de 12,50–2000 ng/mL.

### Materiales

Se diseñó un protocolo de validación empleando tanto materiales de control de calidad suministrados por el fabricante (Control 1 y 2), como soluciones de muestras de líquido pleural.

Se recogieron 20 muestras de líquido pleural en tubos de heparina sódica mediante toracocentesis, y se enviaron inmediatamente al laboratorio, donde se centrifugaron a 415×*g* durante 5 minutos. Se separó el sobrenadante para su análisis.

Las soluciones se realizaron mediante la mezcla homogénea de varias muestras de líquido pleural hasta alcanzar la concentración deseada.

### Estabilidad

La estabilidad se evaluó siguiendo las directrices de la Sociedad Española de Medicina de Laboratorio (SEQC^ML^) [10].

En primer lugar, se obtuvieron tres soluciones de muestras con diferentes concentraciones de CP de nueve muestras de líquido pleural (tres muestras por cada solución): solución 1 (350 ng/mL), solución 2 (11,340 ng/mL), y solución 3 (145,525 ng/mL). Todas las soluciones se dividieron en diferentes alícuotas. Tres alícuotas de cada concentración se conservaron a 4 °C, mientras que cinco alícuotas se congelaron a −80°. Las alícuotas refrigeradas se analizaron cada 2 o 3 días a lo largo de una misma semana, mientras que las alícuotas congeladas se analizaron una vez por semana durante 5 semanas. La estabilidad se expresó como la diferencia entre la concentración en cada condición de conservación y su nivel basal correspondiente (desviación porcentual, DP%). La inestabilidad máxima permisible (IMP) se estableció en ±10 %.

### Imprecisión y sesgo

La imprecisión se evaluó siguiendo la guía EP15 del Insituto de Normas Clínicas y de Laboratorio (CLSI) [11]. Se analizaron dos soluciones obtenidas a partir de seis muestras de líquido pleural (tres muestras por cada solución) con concentraciones similares a las de los materiales de control de calidad, siendo el valor objetivo de CP de 607,5 ng/mL y 121,12 ng/mL para los controles 1 y 2, respectivamente. Se analizaron cinco réplicas en el mismo día (imprecisión intradía) durante cinco días consecutivos (imprecisión interdía). También se analizaron los dos niveles de los controles de calidad mencionados anteriormente en tres réplicas durante cinco días consecutivos. Los resultados se expresaron como coeficientes de variación (CV).

El sesgo se calculó en función de los materiales de control de calidad.

### Linealidad

La linealidad se verificó siguiendo las indicaciones de la Guía EP06 de la CLSI [12]. Se diluyó una solución de elevada concentración obtenida a partir de tres muestras de líquido pleural (−10 % del límite superior de cuantificación) con una solución de baja concentración de otras tres muestras de líquido pleural (+10 % del límite inferior de cuantificación) en diferentes proporciones (1, 0,75, 0,5, 0,25 y 0 % de solución de concentración elevada), cubriendo así el rango dinámico de la prueba. Los duplicados de cada nivel se analizaron en un mismo día. La linealidad se evaluó construyendo una gráfica de la desviación porcentual entre los valores medidos y los valores estimados. La desviación de linealidad (ADL, por sus siglas en inglés) se estableció en ± <desviación.

### Capacidad de detección

Se estudiaron los límites de blanco (LoB), detección (LoD) y cuantificación indicados (LoQ) indicados por el fabricante (0,267, 0,9875 y 12,50 ng/mL, respectivamente) siguiendo la guía EP17 del CLSI [13]. Para tal fin, se utilizó el diluyente comercial (“muestras blanco”), así como ocho muestras de líquido pleural con concentraciones muy bajas de CP cercanas al LoD (“muestras de detección”) o al LoQ (“muestras de cuantificación”) indicado por el fabricante.

El límite de blanco se calculó mediante una prueba no paramétrica calculando el percentil 95 de la distribución de los resultados del blanco de muestra. El LoD se calculó como: LoB + Cp×desviación estándar (DE) entre los resultados de la muestra de detección, donde Cp es un multiplicador para dar el percentil 95 de una distribución normal. El LoQ se determinó como la concentración del mensurando en la intersección de la línea de ajuste de un modelo de función potencial (precisión intra-laboratorio, expresada como el coeficiente de variación porcentual con respecto a los resultados de las muestras de cuantificación) con la precisión objetivo de un CV del 10 %.

### Efecto de arrastre

Siguiendo las recomendaciones de la IUPAC de 1991 [14], el efecto de arrastre se evaluó midiendo dos soluciones de muestras de líquido pleural obtenidas a partir de tres muestras de líquido pleural con concentraciones elevadas (a) y otras tres con concentraciones bajas (b) de CP. Se empleó una secuencia de dos alícuotas sucesivas de solución con un valor elevado (a), seguida de una secuencia de tres alícuotas sucesivas con una concentración baja (b). Esta secuencia (a1, a2, b1, b2, b3) se procesó 10 veces en una misma serie.

### Análisis estadístico

El análisis estadístico se realizó con el programa MedCalc^®^ (12.5.0) y Excel (16.70). Las variables continuas se presentaron como medias y DE, mientras que las variables discretas se expresaron en valores absolutos o porcentajes. El estudio de normalidad se evaluó mediante la prueba de Kolmogorov-Smirnov. Un valor p<0,05 se consideró estadísticamente significativo. La comparación de muestras pareadas entre grupos se realizó mediante la prueba de los rangos con signo de Wilcoxon.

### Consideraciones éticas

Este estudio se realizó con ajuste a los principios de la Declaración de Helsinki de la Asociación Médica Mundial, en lo relativo a la realización ética de estudios en humanos. Todos los participantes firmaron un consentimiento informado, en virtud del protocolo aprobado por el Comité Autonómico de Ética de la Investigación (código 2022.245).

## Resultados

### Estabilidad

**Figura 1:**
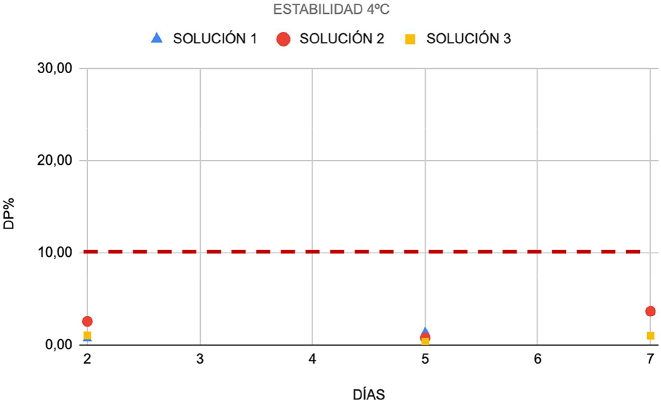
Estabilidad de las soluciones 1, 2 y 3 refrigeradas a 4 °C, expresada como DP (%). DP, desviación porcentual.

**Figura 2:**
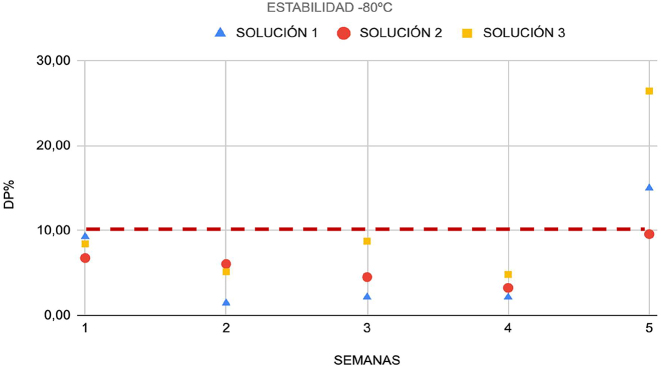
Estabilidad de las soluciones 1, 2 y 3 congeladas a −80 °C expresada como DP (%). DP, desviación porcentual.

La CP permaneció estable durante al menos una semana en líquido pleural refrigerado (Figura 1), y durante cuatro semanas a −80 °C (Figura 2). A ambas temperaturas, la desviación porcentual fue inferior a la inestabilidad máxima permisible (IMP) (10 %).

### Imprecisión y sesgo

La imprecisión intra e interlaboratorio observada fue similar en los materiales de control de calidad interno y en las soluciones de muestras de líquido pleural, oscilando entre el 2,2 % y el 6,5 %. Así mismo, se calculó un sesgo negativo del 3,0 % y 5,5 % (Tabla 1).

**Tabla 1:** Estudio de imprecisión (intradía e interdía) y sesgo. CV, coeficiente de variación; CP, calprotectina.

	CP medida, ng/mL	CV intradía, %	CV interdía, %	Sesgo, %
Solución de líquido pleural de elevada concentración	697,5	5,0	6,5	–
Solución de líquido pleural de baja concentración	153,2	4,7	5,5	–
Control 1 (objetivo 607,50 ng/mL)	574	4,3	4,0	−5,5
Control 2 (objetivo 121,12 ng/mL)	117,5	2,2	5,9	−3,0

### Linealidad

**Figura 3:**
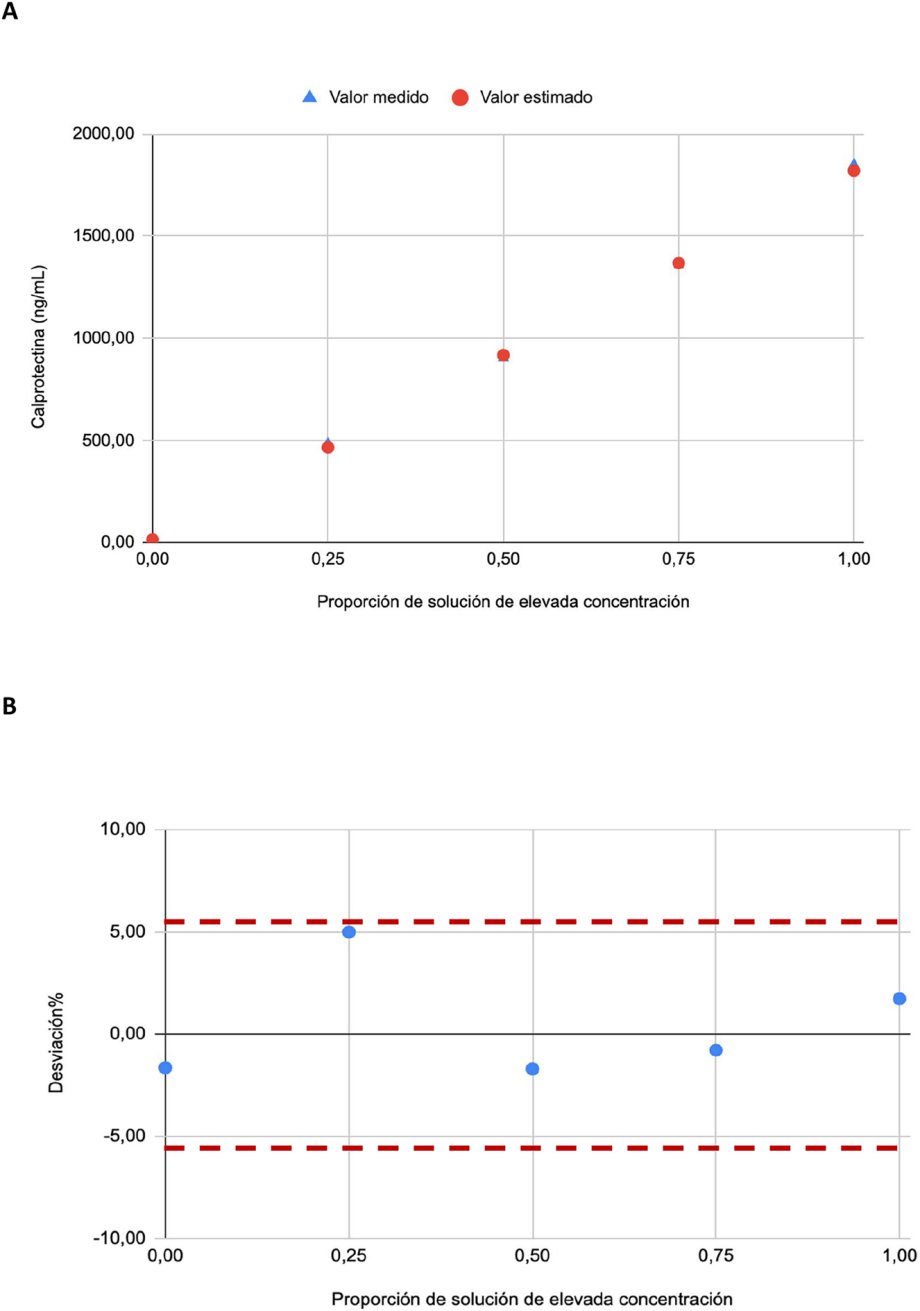
Estudio de linealidad de la prueba Liaison^®^ Calprotectin de Diasorin. (A) Valores estimados frente a medidos. (B) Porcentaje de desviación entre el valor medido y el valor estimado según la proporción de solución de elevada concentración.

La linealidad del método se verificó de 12.5 a 2000 ng/mL (Figura 3). Los ADL observados fueron inferiores al sesgo tolerable en todo el rango de medida.

### Capacidad de detección

El límite de cuantificación (LoQ) de la prueba fue de 48,52 ng/mL. Dado que no se demostró una linealidad inferior a 12,50 ng/mL (6,453 unidades de quimioluminiscencia, RLU), no se pudo calcular el valor de LoB y LoD, siendo ambos aproximadamente cuatro veces inferiores al LoQ.

### Efecto de arrastre

La mediana de los niveles de CP en el grupo b1 fue significativamente superior al grupo b3 [354 (348–360) frente a 339 (332–345) ng/mL, p<0,05]. Se repitió el experimento con valores más bajos de (a) cercanos al límite superior del rango de linealidad, habiendo obtenido [329 (315–330) frente a 316 (315–325) ng/mL, p<0,05] (Tabla 2). Se observó un efecto de arrastre estadísticamente significativo del 3,62 % y 2,92 %, respectivamente.

**Tabla 2:** Resultados del estudio de arrastre en los dos escenarios: niveles muy altos de CP (arriba), y cerca del límite superior de los niveles de linealidad (abajo).

	a1	a2	b1	b2	b3	Efecto de arrastre (valor de p)
Mediana de CP muy alta, ng/mL	10,912	10,962	354	347	338	0,0488
Mediana de CP alta, ng/mL	2,145	2,244	329	320	316	0,0195

## Discusión

En la práctica clínica actual, los niveles de CP se suelen medir únicamente en muestras fecales para el diagnóstico y seguimiento de pacientes con patologías gastrointestinales inflamatorias crónicas [4].

Sin embargo, en los últimos años se ha señalado a la CP como un posible biomarcador para el seguimiento de enfermedad activa en otras enfermedades autoinmunes, ya que la CP ejerce un papel fundamental en la inmunidad innata [15]. De hecho, ya se han descrito varios ejemplos de su utilidad clínica, como la elevación de los niveles de CP en plasma y suero en pacientes con artritis reumatoide (AR) [16, 17] y espondiloartritis axial (axSpA) [18].

La CP se puede encontrar en otros fluidos biológicos, como los derrames. Parece que la medida de CP en fluido sinovial es de interés en pacientes con AR [16, 17], y en líquido ascítico para el diagnóstico de peritonitis bacteriana espontánea en pacientes con cirrosis hepática [19], [20], [21]. Respecto al líquido pleural, la CP participa en numerosos procesos celulares relacionados con la salud y las patologías pulmonares. Además de sus funciones antimicrobianas, la CP también posee propiedades pro y antitumorales relacionadas con la supervivencia y el crecimiento pulmonar, la angiogénesis, la respuesta al daño en el ADN y la remodelación de la matriz extracelular [22]. Recientemente, se ha aportado evidencia de su utilidad a la hora de predecir malignidad [6], [7], [8] y diferenciar entre derrames pleurales paraneumónicos y no paraneumónicos [22, 23]. Sin embargo, los estudios realizados para evaluar los niveles de CP en derrames pleurales son muy escasos y presentan limitaciones, relacionadas con las técnicas de medición empleadas, ya que estas se basan en procedimientos ELISA, a pesar de que, en los últimos años, han aparecido analizadores completamente automáticos. Este es el primer estudio en determinar la calidad analítica de la medición de CP en líquido pleural mediante una prueba de quimioluminiscencia totalmente automática (DiaSorin Liaison^®^).

En el presente trabajo, demostramos que la estabilidad de la CP en líquido pleural (7 días) es incluso superior que en heces (72 horas), y que en muestra de plasma EDTA (4 días) [24] en condiciones de refrigeración.

La imprecisión observada en soluciones de muestras de líquido pleural es inferior a la descrita en el método ELISA (≤8,67 %, ≤12,82 %, intra e interdía, respectivamente) [6], similar a la hallada en nuestros materiales de control de calidad interno, y ligeramente superior a la indicada por el fabricante de las muestras fecales (≤3,10 %, ≤4,00 %, intra e interdía, respectivamente). Observamos un sesgo negativo en los materiales de control de calidad interno que se mantiene dentro del margen de imprecisión del método. De este modo, se podría emplear dicho material de control en futuros estudios en los que se emplee líquido pleural como matriz, considerando introducir dicha matriz en el prospecto del fabricante.

La linealidad declarada se ha verificado en LP.

Observamos un LoQ ligeramente superior en el líquido pleural que en las heces, lo que se debería tener en cuenta para futuras aplicaciones donde las concentraciones bajas de CP sean de interés clínico. Aún así, el LoQ de este método es inferior al descrito en ELISA (ELISA fCAL^®^) o en el método inmunocromatográfico de flujo lateral (Quantum Blue^®^) (400 y 5,000 ng/mL respectivamente) [8].

Finalmente, se puede observar un ligero efecto de arrastre no solo al medir concentraciones muy elevadas de CP, sino también cerca del límite superior del rango de linealidad. No obstante, al encontrarse aún dentro de los márgenes de tolerancia de calidad analítica, el efecto de arrastre no debería producir diferencias clínicamente relevantes.

En conclusión, el presente estudio demuestra que la prueba de quimioluminiscencia automática tiene una mejor calidad analítica que otros métodos descritos en la literatura.

## Conclusiones

La prueba Liaison^®^ Calprotectin de DiaSorin permite medir la CP con precisión en líquido pleural, y la calidad analítica aquí descrita abre una vía para futuras investigaciones sobre su posible utilidad en la práctica clínica.
